# Single-photon-multi-layer-interference lithography for high-aspect-ratio and three-dimensional SU-8 micro-/nanostructures

**DOI:** 10.1038/srep18428

**Published:** 2016-01-04

**Authors:** Siddharth Ghosh, G. K. Ananthasuresh

**Affiliations:** 1 Department of Mechanical Engineering, Indian Institute of Science, Bangalore, 560012, Karnataka, India; 2 Third Institute of Physics, Georg-August-Universität-Göttingen, Friedrich-Hund-Platz-1, Göttingen 37077, Germany

## Abstract

We report microstructures of SU-8 photo-sensitive polymer with high-aspect-ratio, which is defined as the ratio of height to in-plane feature size. The highest aspect ratio achieved in this work exceeds 250. A multi-layer and single-photon lithography approach is used in this work to expose SU-8 photoresist of thickness up to 100 μm. Here, multi-layer and time-lapsed writing is the key concept that enables nanometer localised controlled photo-induced polymerisation. We use a converging monochromatic laser beam of 405 nm wavelength with a controllable aperture. The reflection of the converging optics from the silicon substrate underneath is responsible for a trapezoidal edge profile of SU-8 microstructure. The reflection induced interfered point-spread-function and multi-layer-single-photon exposure helps to achieve sub-wavelength feature sizes. We obtained a 75 nm tip diameter on a pyramid shaped microstructure. The converging beam profile determines the number of multiple optical focal planes along the depth of field. These focal planes are scanned and exposed non-concurrently with varying energy dosage. It is notable that an un-automated height axis control is sufficient for this method. All of these contribute to realising super-high-aspect-ratio and 3D micro-/nanostructures using SU-8. Finally, we also address the critical problems of photoresist-based micro-/nanofabrication and their solutions.

Polymeric high-aspect-ratio micro-/nanostructures have wide range of applications in the field of micro-/nanosystems, biomechanics and opto-electronics^1^,^2^,^3^,^4^,^5^. As a fabricating material, photopolymers are useful not only for patterning semiconductors, but also for fabricating micro- and nanostructures directly out of them^6^. One of the widely used and inexpensive photopolymers in micro-/nanofabrication research and industry is SU-8. Several groups have reported high-aspect-ratio SU-8 microstructures with different techniques^7^,^8^,^9^,^10^,^11^. Among them femto-second pulsed laser lithography, commonly known as *Direct Laser Writing*, produces finer results as compared to typical mask-photolithography^12^,^13^. The concept of multi-photon lithography yields nanoscale features with different photosensitive polymers^12^. Two things make this possible. The first is the femtosecond pulsed laser source induced high photon flux density, and the second is the precision of an automated high-resolution three-axis piezoelectric scanning stage^12^. However, fabricating specifically free-standing and un-collapsed high-aspect-ratio polymeric micro-/nano-structure still remains a challenge.

In this paper, we introduce a multi-layer and single-photon lithography process to obtain high-aspect-ratio micro-/nanostructures. Along with multi-layer and single-photon lithography technique, we demonstrate an interfered point spread function (PSF) that was generated using incident and reflected laser beam from silicon surface underneath the SU-8 film. Such interfered PSF was also used by Schnitzbauer *et al.*^14^ in a modified 4-π fluorescence microscopy to achieve high axial resolution of Stefan Hell developed typical ‘Double-confocal scanning microscopy^15^. Fabrication of micro-/nanostructures with aspect ratios exceeding 250:1 is presented here. Such high-aspect-ratio is obtained with nanometer scale structural feature. For example, 205 nm in-plane feature size was obtained when the aspect ratio was 200:1. Considering photolithographic technique, such nanoscale feature size is very small. These kinds of high-aspect-ratio micro-nanostructures are immediate interests of different biomechanical and nano-optical investigations^16^,^17^. For higher aspect-ratio than 200, we report a feature size of 75 nm at an AFM probe like sharp tip-end, which is applicable for near-field scanning optical microscopy (NSOM) of single molecules and nanoparticles^18^,^19^,^20^.

Beside the lithography process, some critical issues were overcome, without which demonstration of narrow and high-aspect-ratio microstructure is not possible. We discuss some problems with direct-laser-writing such as photo-activated thermal swelling, stitching error and interacting reflected beams^2^,^21^,^22^. The material and physical properties of the photoresist such as solvent content and thermal expansion coefficient also influence the ultimate microstructures. Another critical problem lies in post-development drying; superfine free-standing tall structures often collapse. This problem is because of the high vapour pressure of the developer being used^2^,^22^,^23^. In addition to collapse, in many instances cracking of collapsed SU-8 microstructure is observed. This is due to capillary condensation along with high vapour pressure of the developer^23^. For high-aspect-ratio microstructures, structural rigidity should also be taken into account. If the structure is superfine and extremely tall, it cannot sustain the development and consecutive conventional drying process - leading to its collapse. In short, Table 1 enlists these problems, which we have encountered and their solutions.

## Theory

### Single-photon multi-layer interference lithography

The lowest energy absorbed by SU-8 monomers is of 400 nm wavelength. At this wavelength, single photon absorption polymerises multiple SU-8 monomers by diffused photo-acid generation^7^. So, we used slightly lower energy photons as compared to its lowest absorbable energy to avoid such unwanted polymerisation resulting to bigger feature sizes than intended. We use a multi-layer approach to provide sufficient polymerising energy along the thickness of the SU-8 film, which will be the ultimate height of any high-aspect-ratio micro-/nanostructure. We also use an interfered PSF to avoid unwanted polymerisation by reducing energy distribution along the height axis of the PSF as compare to typical confocal PSF. We obtain nanoscale feature by exposing certain regions along the height axis with partially interfered photons of the PSF. Considering all the above facts, we name this photolithography process as single-photon multi-layer interference lithography.

### Reflected interfered point spread function

**Figure 1:**
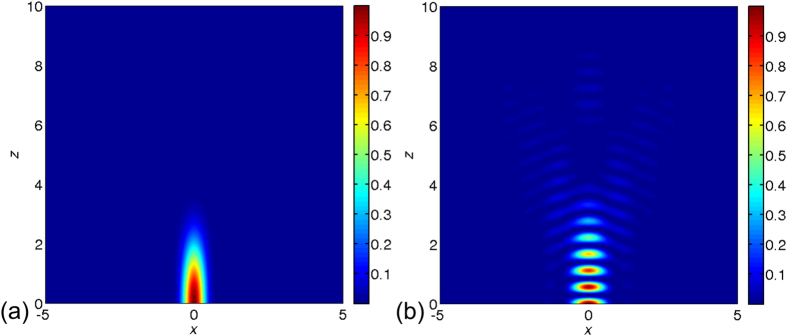
(**a**) Confocal PSF and (**b**) interfered PSF of incident and reflected beam inside an SU-8 thin-film (n_SU-8_ = 1.66). In all PSFs the *x* and *z* axis has μm units and maximum intensity is at (0, 0) position, which is silicon-SU-8 interface.

**Figure 2:**
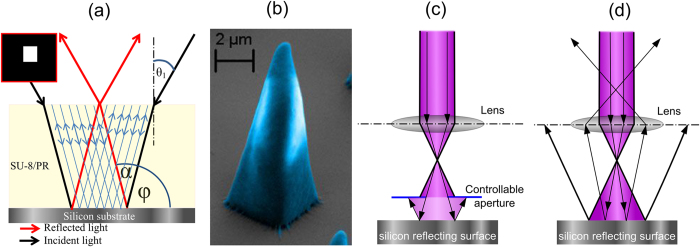
(**a**) Schematic of single layer focusing and resulting aslant edge profile (for a given square shaped design). (**b**) Pyramidal microstructure created due to single layer focusing. (**c**) Shows the usage of aperture for writing a certain dimension and (**d**) shows multiple reflection and need for controllable opening (also used for variable specified design parameters)^22^.

A converging confocal monochromatic laser beam of 405 nm is reflected back into the resist from a nanometer surface roughened silicon surface. Such reflection interacts with the incident beam and due to interference a PSF is generated, which is different from typical confocal PSF. In Fig. 1 we compare such interfered PSF with a typical confocal PSF. The PSFs are simulated considering the reflection from a silicon surface inside an SU-8 thin-film. Here, the SU-8 is considered by using a refractive index of 1.66. We simulated the PSFs using fast Fourier transformation of the pupil function^14^. A typical confocal PSF on a silicon surface is plotted in Fig. 1(a). On contrast to that the reflected PSF interfering with the incident beam is shown in Fig. 1(b). In both the plots the focusing position is at the interface of silicon and SU-8. Such reflected PSF produces a smaller interrupted energy distribution along the z-axis, which produces finer polymerising region as compared to typical confocal PSF. A trapezoidal edge profile can be achieved when this kind of reflected interfered PSF is used to polymerise SU-8 thin-film. If the thickness of the photoresist is large enough to accommodate the convergence, a pointed tip can be achieved in nanometer scale. This phenomenon can be modified and applied as a useful tool to realise uniform cross-sectional high-aspect-ratio microstructures. We use the reflection principle in layer by layer approach by focusing laser at different depths of field to produce uniform vertical edge profiled microstructures. This kind of multi-layer approach is known as optical sectioning in 3D tomographic imaging^24^,^25^ Here, a light beam is able to focus at different objective-planes/optical planes along the depth of the SU-8 thin-film. The total number of planes is dependent on the numerical aperture^24^. This concept is schematically illustrated in Fig. 2. In Fig. 2(a), a refracted converging light beam with incident angle *θ*_*1*_ reflects from the substrate-photoresist interface with reflected angle *α*, and produces an aslant edge profile with an exterior angle *α* + *ϕ.* This aslant edge profile is evident with a polymerised SU-8 microstructure in Fig. 2(b). Here, the reflected photons interact with the other reflected photons. This phenomenon is shown in Fig. 2(a), where red paths are the reflected beams, which interact with each other for a given 2D square shaped design to write in 3D. The blue paths in Fig. 2(a) are the interacting photons. This interaction creates more energy as compared to a single or parallel beam, which does not converge with the other beams. The reflected interfered PSF leads to partial and fully destructive interference and helps to obtaining superfine sub-wavelength features. Using this principle a trapezoidal edge profile can be observed in Fig. 1(b). Further, such a pyramidal structure allowed us to proceed towards modifying the optical energy scheme in achieving uniform cross-sectional microstructures. Here, the limitation of producing the superfine feature is also dependent on a controllable aperture as illustrated in Fig. 2 (c,d). Figure 2(d) shows the significance of controllable aperture, where absence of which results to multiple reflections. Here, we used an optical bench routine called Physlets to calculate the aperture diameter and its position^22^. The total optical setup is presented in supplementary information (Fig. S1(c)).

### Multi-layer writing

**Figure 3:**
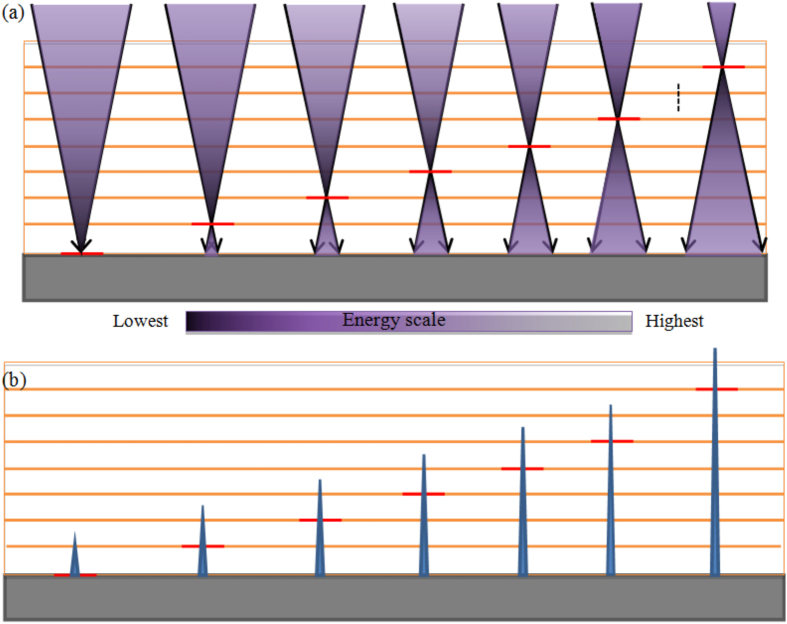
(**a**) Graphical representation of multi-layer writing and (b) resulting polymerised microstructure layer by layer depending on writing focal plane.

**Figure 4:**
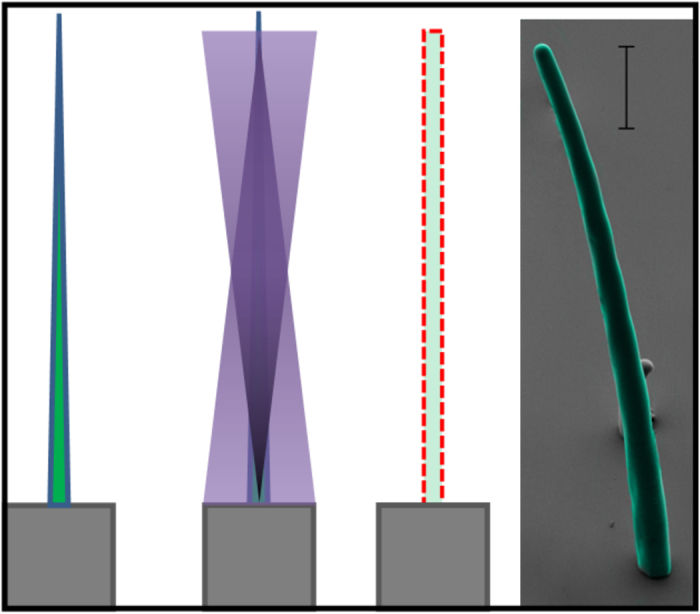
Formation of uniform cross-sectional high-aspect-ratio structure with variable dosage. Here, scale is 20 μm.

**Figure 5:**
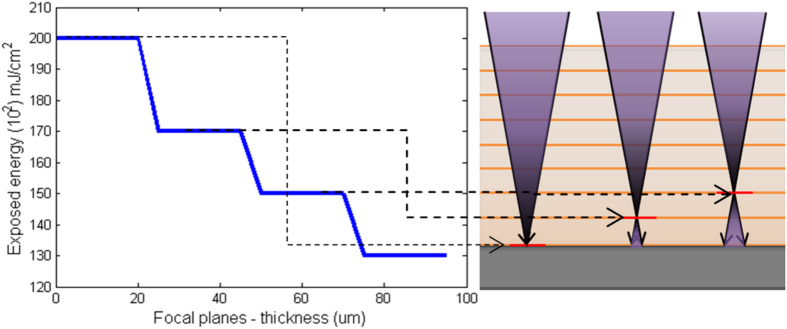
Decreasing energy dosage variation along the height of the SU-8 film thickness.

Here, we used more than 100 μm thick spin-casted SU-8 films to produce high-aspect-ratio microstructures. Scan-exposing photonic energy (writing) only once on such film thickness may not reach the required amount of total energy needed to polymerise the photoresist along the thickness. This problem can be resolved by writing multiple times to ac achieve hieving total required energy of polymerisation. Also to avoid a slant or trapezoidal edge profile and to achieve the required polymerising total energy, multi-layer writing was needed. Additionally, a uniform distribution of energy is required along the total thickness of the photoresist. Figure 3(a) shows the schematic of multi-layer writing by focusing the optics at different objective-planes inside an SU-8 film and thereby ensuring the growth of a polymerising microstructure at each layer, as illustrated in Fig. 3(b). We start the writing from the interface of substrate-photoresist (0 μm height in Fig. 4) and end at the top surface of the photoresist (100 μm height in Fig. 4) after writing all the intermediate objective-planes. If an equal amount of photonic energy is provided to all the layers, the total exposed energy at the bottom will be more than the top surface. So, the structure will be stiffer at the base than the top and results in bending as shown in Fig. 5(a). This is because in multi-layer writing, while writing on any layer the formerly exposed layer is also exposed once again with a low energy defocused diverging beam as schematically explained in Fig. 5(b). However, if we follow the energy scheme of Fig. 4, the microstructure polymerises uniformly and achieves a uniform stiffness along the height as shown in Fig. 5(c,d). The exposure in each layer is compensated by decreasing the energy of exposure at the focal spot along the thickness of the SU-8 thin-film following the aforementioned exposed energy scheme in Fig. 4.

## Results and Discussion

### 3D micro-/nanostructures-Design parameters and multi-layer energy exposure

**Figure 6:**
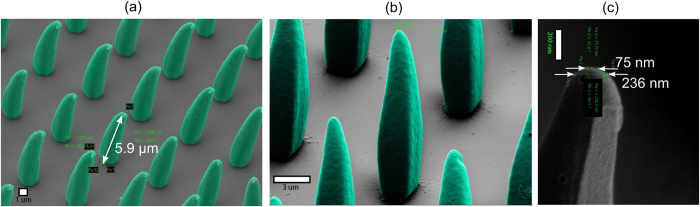
(**a**) The quadrilateral pyramidal microstructure formed due to converging optical reflection (scale bar of 1 μm), (**b**) at lower magnification with 253 nm tip diameter (scale bar 3 μm) and (**c**) at higher magnification with 75 nm tip diameter (vertical scale bar is 200 nm).

The optical reflection of converging monochromatic beams incident on the silicon surface produces pyramids. The design used here for patterning was 1 μm × 1 μm square. Here, the beam was focused at the interface of photoresist and silicon substrate. The SU-8 film thicknesses were around 10 μm. So, to achieve the ultimate energy of exposure six single-plane steps were needed. Here, the obtained tip diameter was 75 nm, where all the four edges converge at a single point (Fig. 6(a–c)).

### Uniform cross-sectional features

**Figure 7:**
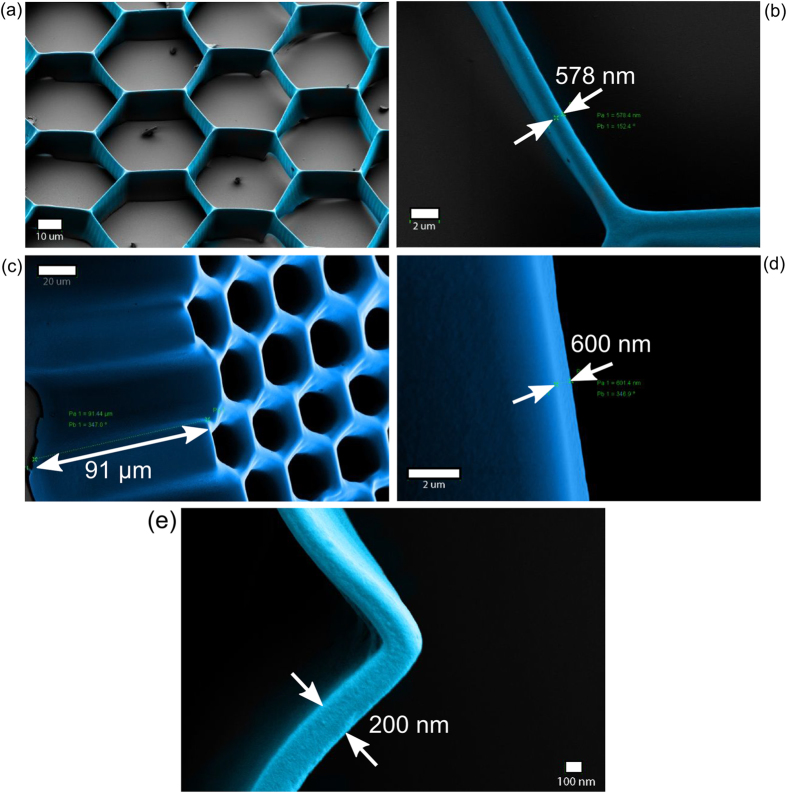
(**a**) The hexagonal features on 10 μm SU-8 film thick ness with (**b**) width of 578 nm, here respective scale bar are 10 μm and 2 μm. (**c**) The hexagonal microstructure with height of 91 μm (scale bar of 20 μm) and (**d**) wall width of 600 nm (scale bar of 2 μm). (e) Another hexagonal 50 μm tall structure with 200 nm wall width (scale bar of 100 nm).

After obtaining sub-wavelength 3D microstructure, uniform cross-sectional features were attempted. Here, multi-layer writing was introduced. This gave rise to uniform cross-sectional features. As a bio-mimetic model of hexagonal hepatocyte cells, a hexagonal structure with a height of 10 μm and width of 578 nm was fabricated (Fig. 7(a,b)) using six successive multi-layer writing. Each plane was written six times following the energy scheme of Fig. 4. The obtained pattern in Fig. 7(a,b) is the same as the given input design, i.e., no distortion is observed. The width kept constant in the design was 500 nm. The uniformity along the cross-section (z axis) is distinctly visible here. Thereafter, we worked with high thickness of photo-resist. Fig. 7(c) shows a ~100 μm tall hexagonal microstructure with a wall width of 500 to 600 nm (Fig. 7(d)). The aspect ratio is nearly 200:1. Another set of hexagonal microstrutres were fabricated with wall width of 205 nm and height of 56 μm, where the aspect-ratio was 273:1 as shown in Fig. 7(e). Thus, irrespective of the thickness of the photoresist film or height, in-plane structural features of less than a micron are achievable using this technique. This kind of high-aspect-ratio hexagonal micro-/nanostructure arrays are useful for scaffolding hexagonal cells such as hepatocytes.

### Nanoscale sub-wavelength features

Another feature obtained here using SU-8 was 75 nm at the tip of a pyramidal shaped AFM like probe. Haske *et al.,* (2007) reported that 65 nm structure is possible with two-photon lithography^26^. The resist used to obtain 65 nm features was 4,4′-bis(di-n-butylamino)biphenyl (DABP) and E,E-1,4-bis[4-(di-nbutylamino)styryl]-2,5-dimethoxybenzene (DABSB), which photo-polymserises at 520 nm and 730 nm of photons, respectively^26^. However, using SU-8 nearly 75 nm of feature size with single-photon absorption of 405 nm laser is not reported to the best of our knowledge. The 75 nm features at the tip are reproducible as evidenced in Fig. 6. The single-layer lithography producing the pyramidal microstructure is also highly reproducible.

### Problems of high-aspect-ratio micro-/nanolithography

Deubel *et al.,* (2004) achieved feature size of 180 nm with SU-8 using multi-photon lithography^12^. They used a high-resolution three-axis piezoelectric scanner^12^. With that they could achieve an aspect ratio of 2.5:1. In our case, the automatic *z* or thickness axis control was not available to be synchronised with a given 3D CAD design. The LW405 used here is a 2D patterning system. So, the laser beam scanner was only synchronized with *x* and *y* axis not with the *z* axis. Our given input here is always a 2D CAD design. We manipulate the *z* axis manually with computer input to the hardware. In our earlier report, we had acheived aspect-ratio of 30:1^13^. There we had compared the single-photon multi-layer lithography with typical mask-photolithography as well as with e-beam lithography^13^. Earlier, we could not cross the diffraction limit due to certain reasons that we have discussed next.

### Number of objective planes and writing speed

**Figure 8:**
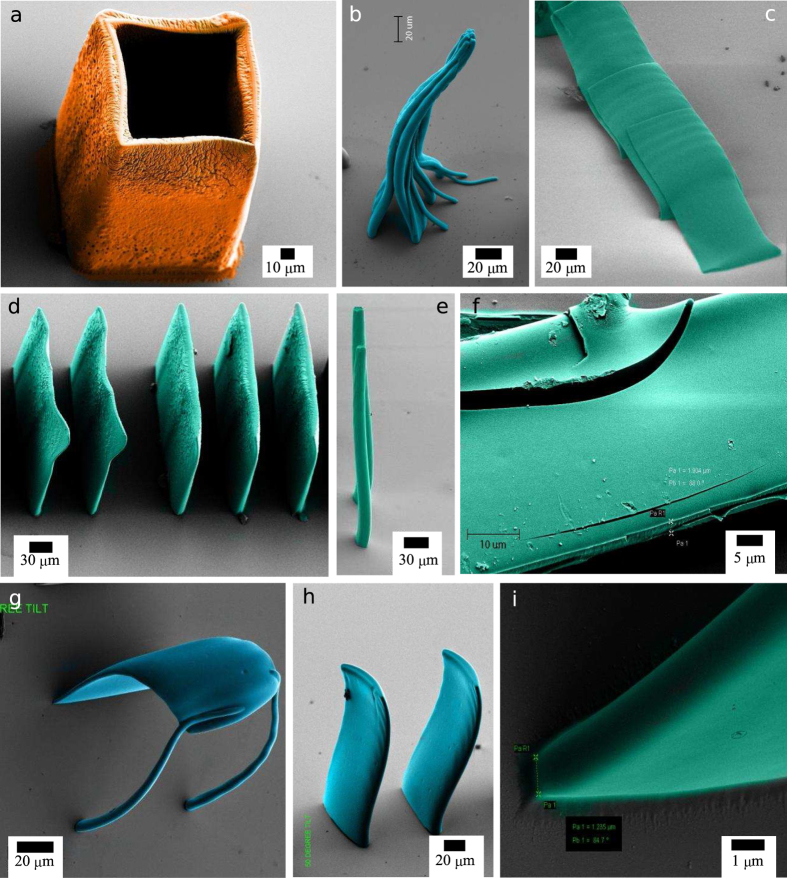
(**a**) 120 μm tall microstructure with over-exposure due to that wall thickness has increased along with cracking and slight distortion (scale bar is 20 μm). (**b**,**c**) Collapsed high-aspect-ratio microstructure (both the scale bars are 20 μm). (**d**,**e**), Stiff microstructure preventing collapse, exposed with more energy than required. Here respective scale bars are 30 μm and 5 μm. (**f**) Cracking due to capillary condensation (scale bar is 10 μm). (**g**) Typical collapsing of SU-8 high-aspect-ratio microstructure (scale bar is 20 μm). (**h**) microstructure preventing collapse with slight distortion (vertical scale bar is 20 μm). (**i**) Good adhesion and uniform adhesion at the base of the microstructure (scale bar is 1 μm).

Empirical results suggest that a 5 μm thickness of focal plane is sufficient to produce uniform cross-sectional microstructures with single layer writing. So, 20 objective-focal-planes were used for 100 μm thickness. Here, each plane was written six times with the energy scheme shown in Fig. 4 to produce uniform cross-sectional high-aspect-ratio features. The theoretical limit of the total number of focal planes, which is dependent on the numerical aperture, does not affect the high-aspect-ratio microstructures. Lastly, to achieve a high quality high-aspect-ratio microstructure, writing speed was fine-tuned to 320 μm/s. Slow writing always leads to over-exposure. This ultimately gives rise to an increase in the size of the intended microstructures, along with cracking (Fig. 8(a)). On the other hand, increasing the writing speed leads to stitching error. The stitching errors are evidenced in supplementary information in Fig. S2 (a–c). By stitching error we mean, when two writing strips cannot keep a correlation with each other and generates an unintended mismatch at the transition of the two strips. This kind of stitching error occurs when the writing speed is too high for certain thicknesses. Stitching error provides loss of energy at the transition between two strips. Fast writing also affects the controller by miss-matching between two adjacent pixels along the *x* axis. In the supplementary section an array of microscale Velcro structures are affected due to the stitching error (Fig. S2).

### Partial polymerisation

Another problem is partial polymerisation. At the initial stage of our research, we came across the problem of un-polymerised or partially polymerised SU-8. The un-polymerised SU-8 are fine nano-fibers shown in supplementary section (Fig. S3 (a–b)). These are generally not visible unless supercritically dried. We generally do not see the network of un-polymerised SU-8 under SEM since most of the time in random drying they form smooth thin-film coating and helps to merge with adjacent structures due to capillary adhesion. So, proper dosage is to be applied to avoid SU-8 nano-fiber formations. These nanofibers are underexposed polymerised SU-8 molecules, which have high surface energy due to their nanoscale surfaces. Therefore, capillary forces are generated at the nanofiber containing region while developing the microstructure. This is the reason behind common collapsing of SU-8 microstructure.

### Adhesion energy and over-exposure

When the partial polymerisation problem is eliminated with sufficient exposure (Fig. 8a), then also we observed collapsing of structure Fig. 8(b,c,g). The micropillars of Fig. 8(b) are distinctly fabricated, they are fully polymerised and they are uniform along the height. The same holds for Fig. 8(c,g). These structures received total energy needed to complete polymerisation. Here, two concepts work together; one is adhesion energy^23^ and the other is stiffness. The adhesion energy due to bulk capillary force and surface tension^27^ assist to collapse a structure. This happens, if the microstructure’s total stiffness fails to resist the fluidic physical forces while developing. SU-8 structure cannot reach its native modulus of elasticity unless total exposed optical energy is slightly larger than the exact energy required to completing the polymerisation. The same occurred for free standing micro-plates in Fig. 8(d,e,h) with slight distortion. They have not collapsed but slight distortion is observed due to surface tension and high vapor pressure of the developer. The structure is just polymerised and a slight over-exposure can avoid this situation. But if it is highly overexposed such as in Fig. 8(a), cracked and over-scaled surface can be observed.

When, the structures are sufficiently rigid with optimal exposure they need to undergo supercritical CO_2_ drying of the isopropanol to avoid any slight distortion they experience. We should keep in mind; the intended structural design should be sufficiently rigid to withstand all the fluidic external forces. To avoid all the above possibilities, we have designed a closed network of honeycomb as shown earlier. And after super-critical drying, the structure with aspect-ratio of 200:1 and 270:1 both are standing upright without any distortion.

The last and quite frequently occurring problem is cracking of collapsed and dense designed structure as shown in Fig. 8(f). This occurs because of capillary condensation. It is not uncommon that the micro-structure has possibilities of trapping the developer solution and cleaning solvent of high vapor pressure inside certain region. This trapped fluid produces high pressure on the microstructure. Ultimately, this leads to cracking of the whole structure especially when it is kept under high vacuum for electron microscopy. It is also observed the structures sometimes detached from the substrate due to the same reason. A supercritical drying process helps to avoid the capillary condensation as evident in Fig. 8(i). Besides, our lithographic technique provides uniform and sufficient optical energy to the thick SU-8 film. Figure 8(i) shows that at the base of a free-standing microplate, it is well adhered to the silicon substrate. This infers that a sufficient exposure and supercritical drying was performed on a high-aspect-ratio microstructure to sustain the ultra-high-vacuum force within the electron microscopy chamber.

## Conclusions

Here, the single-photon-multilayer interference lithography has produced some fine nanostructures using SU-8 photoresist. The intereferece, multi-layer, and thermal relaxation approach has produced microstructures with aspect-ratios up to 273:1. Most of the feature sizes reported here are below or at sub-wavelength scale of the used photon. Such high-aspect-ratio microstructures with nanometer feature sizes are highly reproducible. We demonstrated one of the highest aspect-ratios with feature sizes below the diffraction limit of optics (~200 nm = λ/2). We have achieved tip diameter of ~75 nm (λ/5.4) and frequently 200 to 500 nm feature sizes are obtained. To the best of our knowledge, with optical lithography using SU-8, this kind of sub-wavelength features along with such high-aspect-ratio are not reported before. Fabricating high-aspect-ratio microstructure involves a lot of difficulties, which are covered here in detail. The hexagonal microstructures are similar to hepatocytes shape. So, the high-aspect-ratio honeycomb network will be employed as hepatocyte scaffolding in near future and can be widely used for biomechanical or wave-guided optical studies. One of the major applications of the nanometer sized sharp tip is in the field of super-resolution optical microscopy for nanoscale imaging of biomaterials towards near-field scanning optical microscopy (NSOM) and wave-guided atomic force microscopy (AFM).

## Methods

### Point Spread Function computation

MATLAB Bessel function ‘besselj’ is used to compute the confocal and reflected interfered PSFs. Here, we consider silicon surface as perfect mirror. The SU-8 thin-film is considered by a refractive index as 1.66.

### Substrate processing

Choosing the substrate for SU-8 lithography is crucial. The optical properties of the substrate’s interfacial surface have a significant role. In this work, (100) silicon wafer is used. The substrate is RCA and piranha cleaned to remove organic contaminants and the native oxide. The pristine substrate is then pre-baked at 300 °C for 15 to 20 min before spin-casting a thick layer of SU-8 to evaporate water molecules from the silicon surface. Then, the substrate is cooled down to room temperature to spin-cast SU-8 thin-film.

### SU-8 thin-film preparation

The resist is coated at certain revolutions per minute (rpm) to obtain a uniform film thickness. It is known that the higher the viscosity the higher film thickness. SU-8 2035 used here has a viscosity of 7000 cSt (source: Microchem Corp.; note: 1 cSt =10^−6^ m^2^/s.) The spin-speeds for certain film thicknesses are taken from Ghosh *et al.,* (2012)^13^. After spin-casting, the coated sample is baked prior to exposure to the laser. The pre-baking time should be long enough to evaporate the solvent of SU-8 completely. For 100 μm film thickness, at least 12 hours of baking is required. Sudden ramp-up of temperature creates a shrinking effect on the film; therefore the rise of the temperature should be gradual starting from room temperature and reaching 65 °C and 95 °C. The supplementary information (Table S1) describes more details about the duration of pre-baking for respective film thickness.

After pre-exposure baking, the sample is allowed to cool down and is placed inside the LaserWriter for patterning. Suitable parameters are to be set for the LaserWriter to achieve the intended micro-/nanostructures. The values of these parameters are achieved by conducting several parametric experiments.

### Post-developmental supercritical drying to avoid collapsing and cracking

Processing of Supercritical CO_2_ drying is carried out after developing the microstructure with SU-8 developer and cleaning with the isopropanol alcohol (IPA). Flushing the IPA with several flushes of supercritical fluid is an important step. If the IPA is not properly flushed out of the chamber, the microstructure collapses because of high vapor pressure of IPA or cracks because of capillary condensation, when exposed atmospheric pressure from the drying chamber (see supplementary information).

## Additional Information

**How to cite this article**: Ghosh, S. and Ananthasuresh, G. K. Single-photon-multi-layer-interference lithography for high-aspect-ratio and three-dimensional SU-8 micro-/nanostructures. *Sci. Rep.* **6**, 18428; doi: 10.1038/srep18428 (2016).

## Supplementary Material

Supplementary Information

## Figures and Tables

**Table 1:** Problems of typical direct laser lithography and prospective solutions of them.

Problem with direct laser writing	Solutions
1. Photo-activated thermal swelling and stitching error.	1. Laser exposure duration and manipulation of geometric parameters.
2. Interacting reflected beam related trapezoidal edge profile	2. Multi-layer single-photon lithography.
3. Collapsing of high-aspect-ratio microstructures.	3. Complete removal of solvent from the thin-film of photoresist and super-critical drying.
