# Supplementary material for: Single-photon-multi-layer-interference lithography for high-aspect-ratio and three-dimensional SU-8 micro-/nanostructures
Source: Sci Rep. 2016 Jan 4;6:18428. doi: 10.1038/srep18428 (PMC4698723; doi:10.1038/srep18428)
Supplement: Supplementary Information [file srep18428-s1.pdf]

Single-photon-multi-layer-interference lithography for high-aspect-ratio and three-dimensional SU-8 micro-/nanostructures

Siddharth Ghosh[1,2,*] and G. K. Ananthasuresh[1]

[1]Department of Mechanical Engineering, Indian Institute of Science, Bangalore, 560012, Karnataka, India.

[2]Third Institute of Physics, Georg-August-Universität Göttingen, Friedrich-Hund-Platz-1, Göttingen 37077, Germany.

SUPPLEMENTARY INFORMATION

1. Solvent removal from SU-8 thin-film

Solvent removal from SU-8 spin-coated film is an important step to achieve such high-aspect-ratio. Table S1 lists thickness dependent prebaking duration at consecutive two different temperatures.

Table S1. Pre-baking 1 and 2 temperature and times for different film thicknesses of SU-8.

SU-8	Thickness of Su8 (μm)	Pre- Baking 1 at 65°C (+/-5°C) Time (min)	Pre- Baking 2 at 95°C (+/-5°C) Time (min)
2035	10	10	60
	20	20	90
	50	40	360
	100	60	720

2. Optical scanning exposure lithography.

A Gallium-Nitride solid-state laser diode photonic source was used in this work. It produces photons with 405 nm of λ. A LaserWriter LW405 (Microtech srl, Palermo, Italy) is used for controlling the mentioned parameters and finally to obtain ultra-fine features. These parameters are energy dosage, speed of writing, time-lag, numerical aperture of lens, and focus of plane (depth of field). The tuning of these parameters is carried out by conducting several trials [S1]. The software converts the digital design (2D top view) of the 3D desired pattern on the photoresist into analog machine-level form. It converts a single design into a group of rectangles (Fig. 5(a-b)). Depending on the objective lenses the resolution can be manipulated. The smallest possible rectangle that can be achieved is of the order of 200 nm × 100 nm. The size of the rectangle in the hardware domain can be varied with the numerical aperture of the lens. The aforementioned values of the rectangle can be achieved by using a

numerical aperture of 0.65. All the rectangles situated in a single column (labeled as y-axis) are considered as a single *strip*.

With a piezoelectric scanning stage on which the substrate is mounted, two axes (x and y) are synchronized with the controller, but not the z axis. The laser is synchronized with the controller along with the input design instruction. It patterns only in the y-axis of a pre-determined plane while writing and thus exposing one strip at a time as shown in Fig. 1 (b). So, in the case of multiple dosages and multiple exposures, each strip gets certain time-lag between successive exposures. This time-lag can be varied by altering the velocity of writing (called the *writing speed*) or the speed of the stage in the y-direction. It is mentioned in the product's literature that one pre-set plane can be exposed at a time. So, in the case of a thick photoresist where multi-layer writing is required, the z-stage should be varied and exposed certain amount of energy. A single column of yellow rectangles refers to a strip. Laser writes in the y-direction as shown with the red arrow exposing a single piece of rectangle at a time. After finishing one strip, it reaches to the next strip and so on.

In Fig. S1(c), we illustrated the optical setup of the lithography, where CCD camera, sample stage and controllable aperture are controlled with a computer.

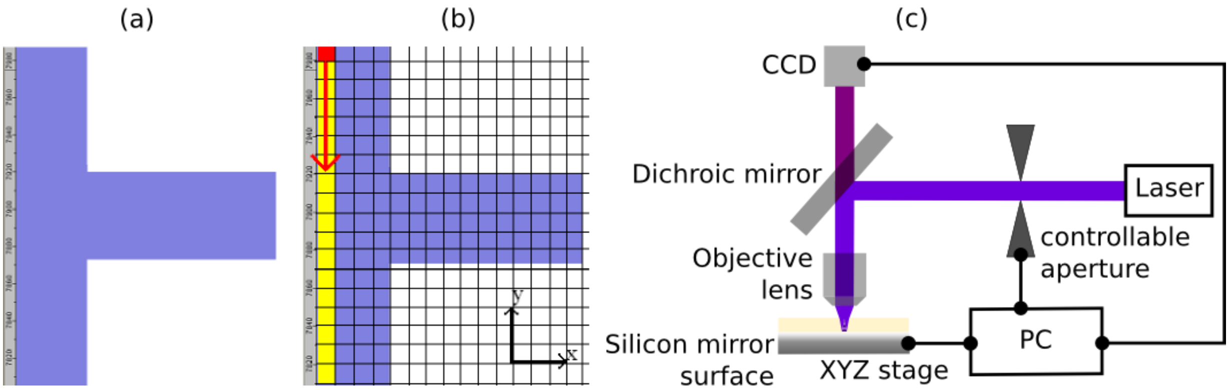

Figure S1. (a) The digital design drawn in AutoCAD and viewed in Clewin in cif format. (b) The schematic of the machine level design after the conversion to analog form [8]. (c) Optical setup used for single-photon multi-layer interfered lithography.

3. Stitching Error.

Fast writing generates stitching error while fabricating arrays of microstructure. Fig. S2 (a-b) shows the collapsed Velcro structure due to stitching error. Due to low-density exposure per unit pixel a line kind of un-intended features come into picture. In Fig. S2 (c) we observe un-collapsed Velcro microstructure, which have slight distortion at the tips. At fast writing speed, the density of raster scanning lines decreases and ultimately line kind of features appear. In Fig. S3 we observed such stitching error when honeycomb microstructures were fabricated at fast scan speed. Fig. S3(a) is a wide view of the honeycomb microstructures and in Fig S3(b) we observed the unintended line kind of features, which primarily contains partially polymerized SU-8 nanofibers as discussed in the next section.

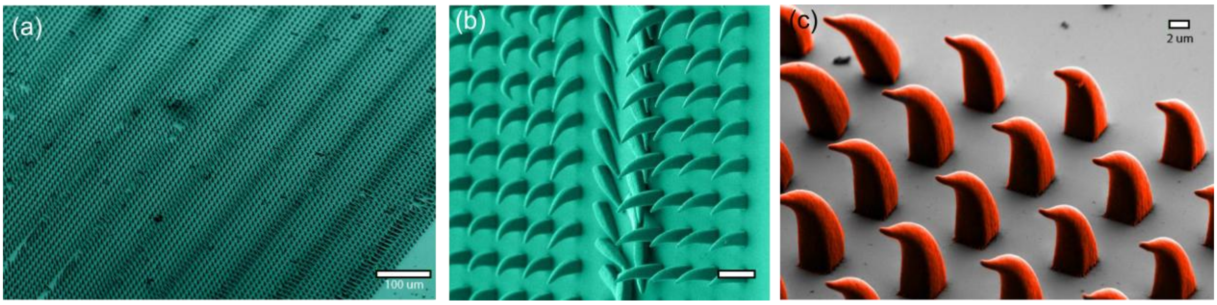

Figure S2. (a) The array of Velcro microstructure with the stitching error at the dark regions (scale bar is 100 µm). (b) One of the dark green arrays of is magnified, where the Velcro microstructures are collapsed due to stitching error regions (scale bar is 10 µm). (c) Magnified view of the Velcro microstructure. Here, the quadrilateral pyramid microstructure is slightly distorted regions (scale bar is 2 µm).

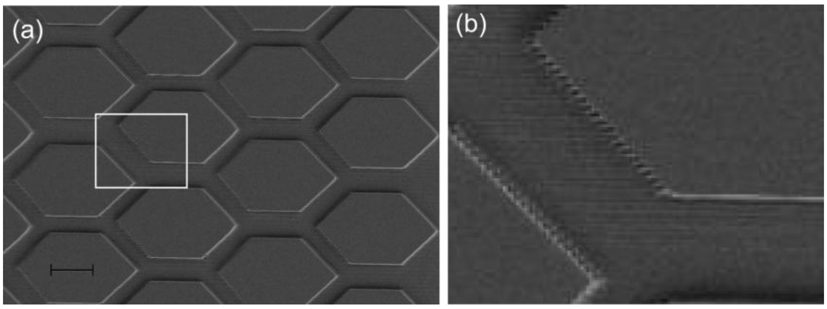

Figure S3. (a) Hexagonal honeycomb microstructure that was written in fast writing speed. (b) At fast writing speed due to stitching error the line kind of features gets prominent in hexagonal honey comb structures.

4. Partial Polymerization and SU-8 nanostructures.

Fig. S4 demonstrates in partial polymerization of SU-8. The blue region is polymerized region and the green region is unpolymerized SU-8 nanofibers. This kind of unpolymerized SU-8 micro-environment helps to collapse adjacent microstructure.

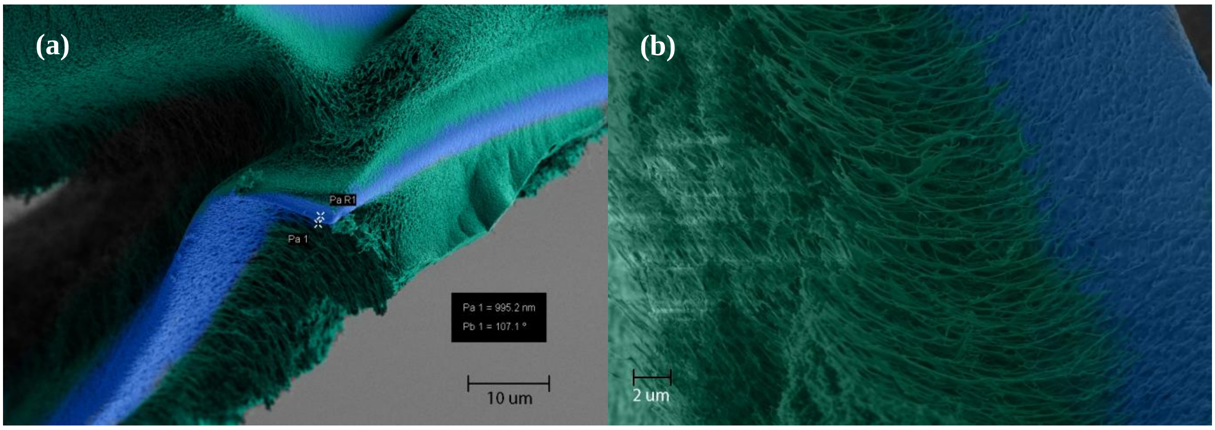

Figure S4. (a) Partially polymerized SU-8 structure. The green portions are unpolymerized and the blue shaded part is polymerized. (b) The nanoscale fibers of unpolymerized SU-8 after supercritical drying.

Reference

S1. Ghosh S. and Ananthasuresh G. K. 2012 A note on high aspect-ratio su-8 micromechanical structures using mask-less direct laser writing, Proc. ASME Int. Man. Sci. Engg. Conf. 2012 MSEC2012
